# Comparative reliability and diagnostic performance of conventional 3T magnetic resonance imaging and 1.5T magnetic resonance arthrography for the evaluation of internal derangement of the hip

**DOI:** 10.1007/s00330-017-5069-4

**Published:** 2017-10-06

**Authors:** A. Chopra, A. J. Grainger, B. Dube, R. Evans, R. Hodgson, J. Conroy, D. Macdonald, Philip Robinson

**Affiliations:** 1 Radiology Department, Leeds Teaching Hospitals, Chapel Allerton Hospital, Leeds, LS7 4SA UK; 2 University of Leeds and NHIR Leeds Musculoskeletal Biomedical Research Centre, Chapel Allerton Hospital, Leeds, UK; 3 Trauma and Orthopaedics Department, Harrogate and District NHS Trust, Harrogate, UK; 4 Trauma and Orthopaedics Department, Leeds Teaching Hospitals, Chapel Allerton Hospital, Leeds, UK

**Keywords:** Femoroacetabular impingement, MR Arthrography, 3T MRI, Acetabular labral tear, Articular cartilage

## Abstract

**Objective:**

To compare the diagnostic accuracy of conventional 3T MRI against 1.5T MR arthrography (MRA) in patients with clinical femoroacetabular impingement (FAI).

**Methods:**

Sixty-eight consecutive patients with clinical FAI underwent both 1.5T MRA and 3T MRI. Imaging was prospectively analysed by two musculoskeletal radiologists, blinded to patient outcomes and scored for internal derangement including labral and cartilage abnormality. Interobserver variation was assessed by kappa analysis. Thirty-nine patients subsequently underwent hip arthroscopy and surgical results and radiology findings were analysed.

**Results:**

Both readers had higher sensitivities for detecting labral tears with 3T MRI compared to 1.5T MRA (not statistically significant p=0.07). For acetabular cartilage defect both readers had higher statistically significant sensitivities using 3T MRI compared to 1.5T MRA (p=0.02). Both readers had a slightly higher sensitivity for detecting delamination with 1.5T MRA compared to 3T MRI, but these differences were not statistically significant (p=0.66). Interobserver agreement was substantial to perfect agreement for all parameters except the identification of delamination (3T MRI showed moderate agreement and 1.5T MRA substantial agreement).

**Conclusion:**

Conventional 3T MRI may be at least equivalent to 1.5T MRA in detecting acetabular labrum and possibly superior to 1.5T MRA in detecting cartilage defects in patients with suspected FAI.

***Key Points*:**

• *Conventional 3T MRI is equivalent to 1.5T MRA for diagnosing labral tears.*

• *Conventional 3T MRI is superior to 1.5T MRA for diagnosing acetabular cartilage defect.*

• *Conventional 3T MRI is equivalent to 1.5T MRA for diagnosing cartilage delamination.*

• *Symptom severity score was significantly higher (p<0.05) in group proceeding to surgery.*

## Introduction

Femoroacetabular impingement (FAI) is a recognised cause of premature osteoarthritis of the hip joint secondary to abnormal mechanical abutment between the proximal femur and acetabular rim [1]. Imaging has a significant role in the early diagnosis of this condition with the hope that early intervention may be able to delay the onset of osteoarthritis. The imaging investigation of choice for suspected FAI is currently MR arthrography (MRA), which has been shown to have greater diagnostic accuracy for detecting labral tears compared to conventional MRI [2]. However, some studies comparing the diagnostic findings of 1.5T MRI with arthroscopy have shown encouraging results for non-arthrographic conventional MRI as a diagnostic tool in FAI [3, 4].

MRA increases contrast resolution making labral and cartilage defects more conspicuous. In a meta-analysis comparing the diagnostic accuracy of MRA and conventional MRI against surgical outcomes in 19 studies, MRA was superior at diagnosing labral tears [2]. However, advances in MRI strength and surface coil technology have necessitated a re-thinking of FAI imaging and the use of conventional 3T MRI instead of MRA is gaining momentum [5]. The main diagnostic challenge for conventional 3T MRI is to identify labral and cartilage lesions with similar sensitivities/specificities to 1.5T MRA.

To the best of our knowledge there have only been three published papers directly comparing both conventional MRI and MRA with arthroscopic findings [6–8]. Of these, the two earliest studies concluded that MRA has superior diagnostic performance over conventional MRI when they compared 1.5T MRA with conventional 1.5T MRI and 3T MRA with conventional 3T MRI, respectively. The most recently published study concluded that conventional 3T MRI was equivalent to 3T MRA for diagnosing labral tears but diagnostically inferior for cartilage lesions [6]. Given the potential to avoid unnecessary intervention, this area of radiology warrants further investigation.

The purpose of this study was to prospectively compare reliability of 1.5T MRA versus conventional 3T MRI in assessing hip FAI-related abnormalities in patients being considered for hip arthroscopy. Moreover, this study aimed to compare diagnostic performance in evaluating FAI-related abnormalities in a subset of patients who underwent hip arthroscopy.

## Materials and methods

### Patient selection and clinical assessment

After institutional ethics approval, symptomatic patients with clinical FAI were recruited into the study by two experienced orthopaedic hip surgeons (authors 6 and 7, 10 and 30 years’ experience, respectively). Patients under the age of 18 years and over the age of 45 years were not included. Symptomatic patients were identified based on a clinical examination protocol [9]. All symptomatic patients had AP and modified frog’s leg radiographs and any patient with previous developmental dysplasia, fracture, inflammatory arthritis or advanced osteoarthritis (Kellgren and Lawrence score 3-4) were excluded from the study. The study lasted 30 months and a total of 68 patients were included in the study, all undergoing informed consent and completing an established hip symptom (for rest and activity) assessment questionnaire prior to imaging: modified Harris Hip (MHH) and Hip disability and osteoarthritis outcome score (HOOS) questionnaires [10–13].

### MR imaging

After recruitment, informed consent and completion of the questionnaires, symptomatic patients underwent 1.5T MRA (total scan time 25 min 11 s) and conventional 3T MRI (total scan time 26 min 3 s) separated by a 3-week period.

#### Imaging protocols

##### 1.5T MR arthrogram

Patients underwent intra-articular injection of 10–15 ml gadolinium solution (gadopentetic acid, dimeglumine, Magnevist 2 mmol/L (Bayer, Leverkusen Germany)) under fluoroscopic imaging with subsequent 1.5T MRI (Siemens Avanto, Erlangen, Germany) using a dedicated large flex surface coil [14]. The MR sequences and their imaging parameters of repetition time (TR), echo time (TE), number of signal averages (NSA) and acquisition times (AT min:sec) are as follows; Coronal T1 fat saturated (FS) – TR 661 ms, TE 11 ms, NSA 2, and AT 5:35, axial T1 FS – TR 781 ms, TE 11 ms, NSA 2, and AT 5:30, sagittal T1 FS – TR 661 ms, TE 11 ms, NSA 2, and AT 5:43, axial oblique T1 – TR 450 ms, TE 11 ms, NSA 2, and AT 3:47 and coronal T2 FS – TR 3,700 ms, TE 81 ms, NSA 1, and AT 4:09. For all sequences slice thickness was 3 mm and pixel size was between 0.52 mm and 0.62 mm.

##### Conventional 3T MRI

3T MRI (Siemens Verio, Erlangen, Germany) with dedicated large flex surface coil; coronal proton density (PD) FS – TR 1,970 ms, TE 23 ms and AT 5:21, axial PD FS – TR 1,970 ms, TE 23 ms and AT 5:21, sagittal PD FS – TR 1,970 ms, TE 23 ms and AT 5:21, axial oblique PD – TR 3,000 ms, TE 31 ms and AT 5:29 and coronal T2 FS – TR 5,000 ms, TE 65 ms and AT 2:12. For all sequences NSA was 1, slice thickness was 3 mm and pixel size was 0.47 mm except for the axial oblique PD (2-mm slice thickness and 0.40-mm pixel size).

### Image analysis

**Fig. 1:**
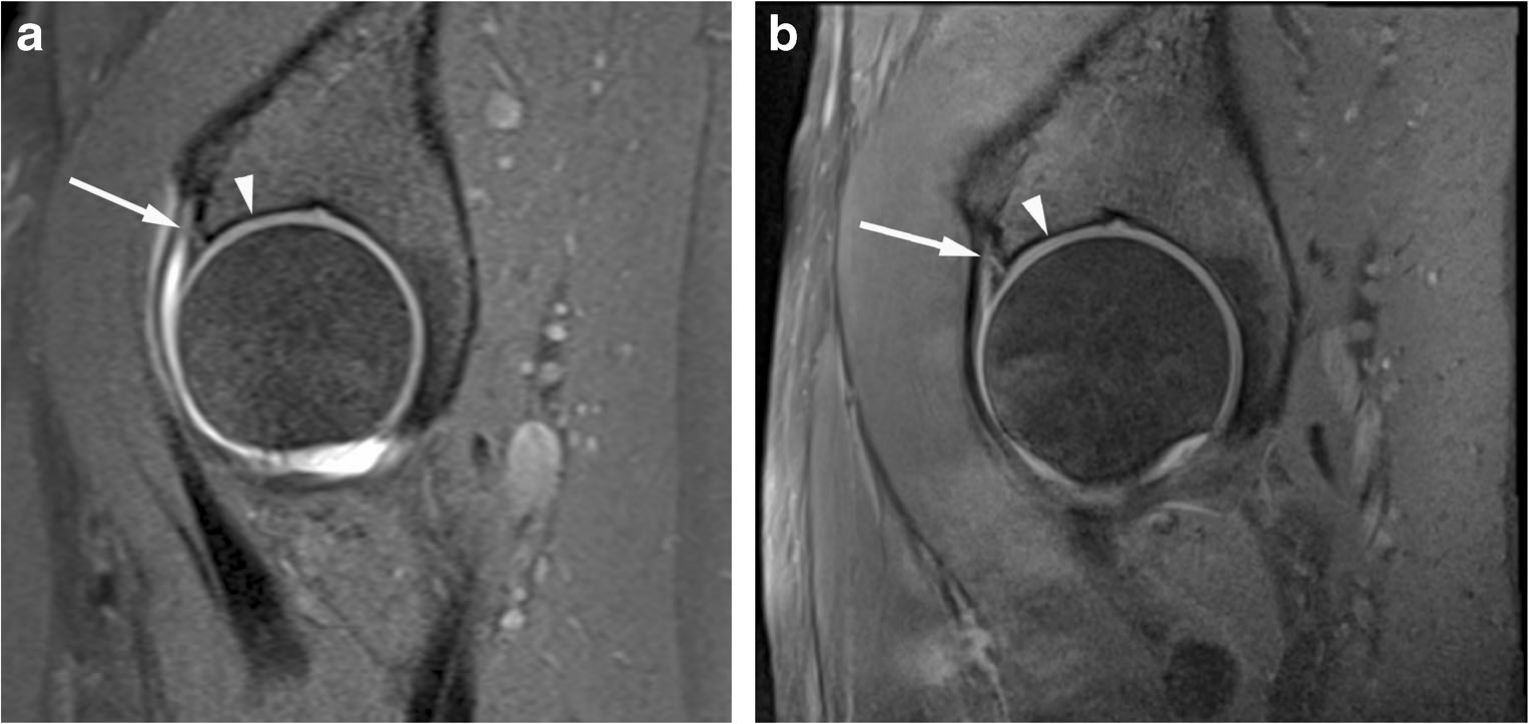
Acetabular labrum complete tear in a 27-year-old confirmed at surgery. Sagittal (**a**) T1 FS 1.5T MRA and (**b**) PD FS 3T MR images show complete basal labral tear (arrow) and normal articular cartilage (arrowhead)

**Fig. 2:**
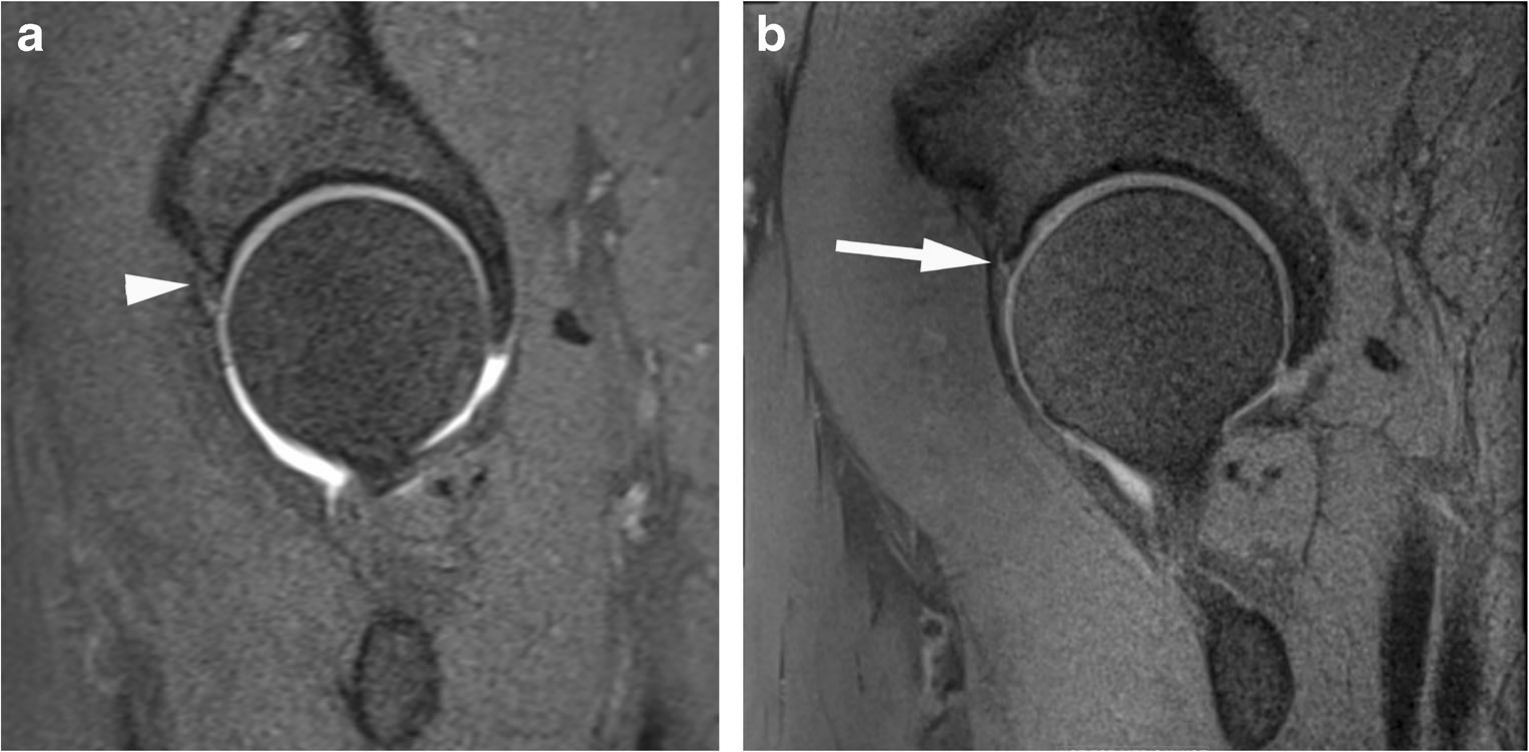
Acetabular labrum complete tear in a 29-year-old confirmed at surgery. Sagittal (**a**) T1 FS 1.5T MRA shows no tear (arrowhead) and (**b**) PD FS 3T MR images shows complete basal labral tear (arrow)

**Fig. 3:**
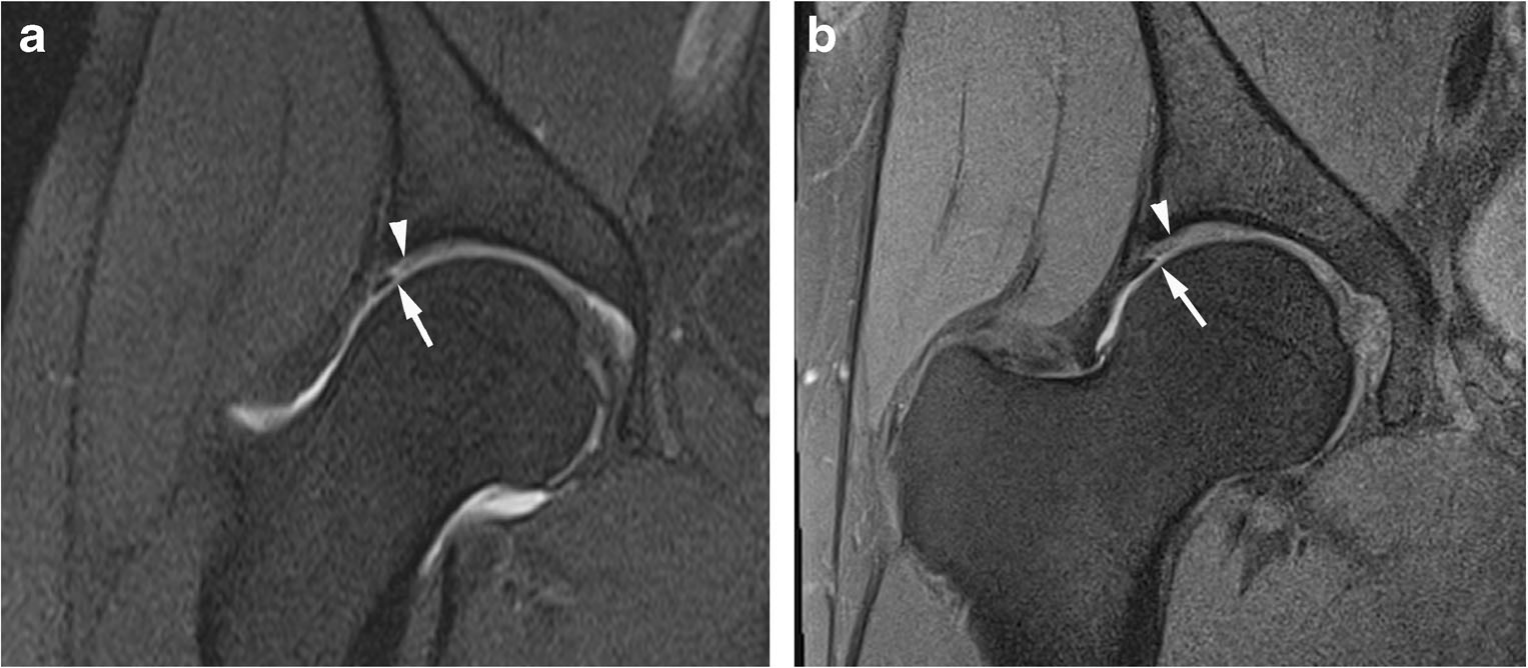
Anterosuperior acetabular labrum partial tear with adjacent cartilage delamination in a 28-year-old confirmed at surgery. Coronal (**a**) T1 FS 1.5T MRA image shows partial labral tear (arrow) and the adjacent cartilage shows increased linear basal fluid signal (arrowhead). (**b**) PD FS 3T MR image shows partial labral tear (arrow) and the adjacent cartilage shows low chondral signal (arrowhead) indicating delamination

**Fig. 4:**
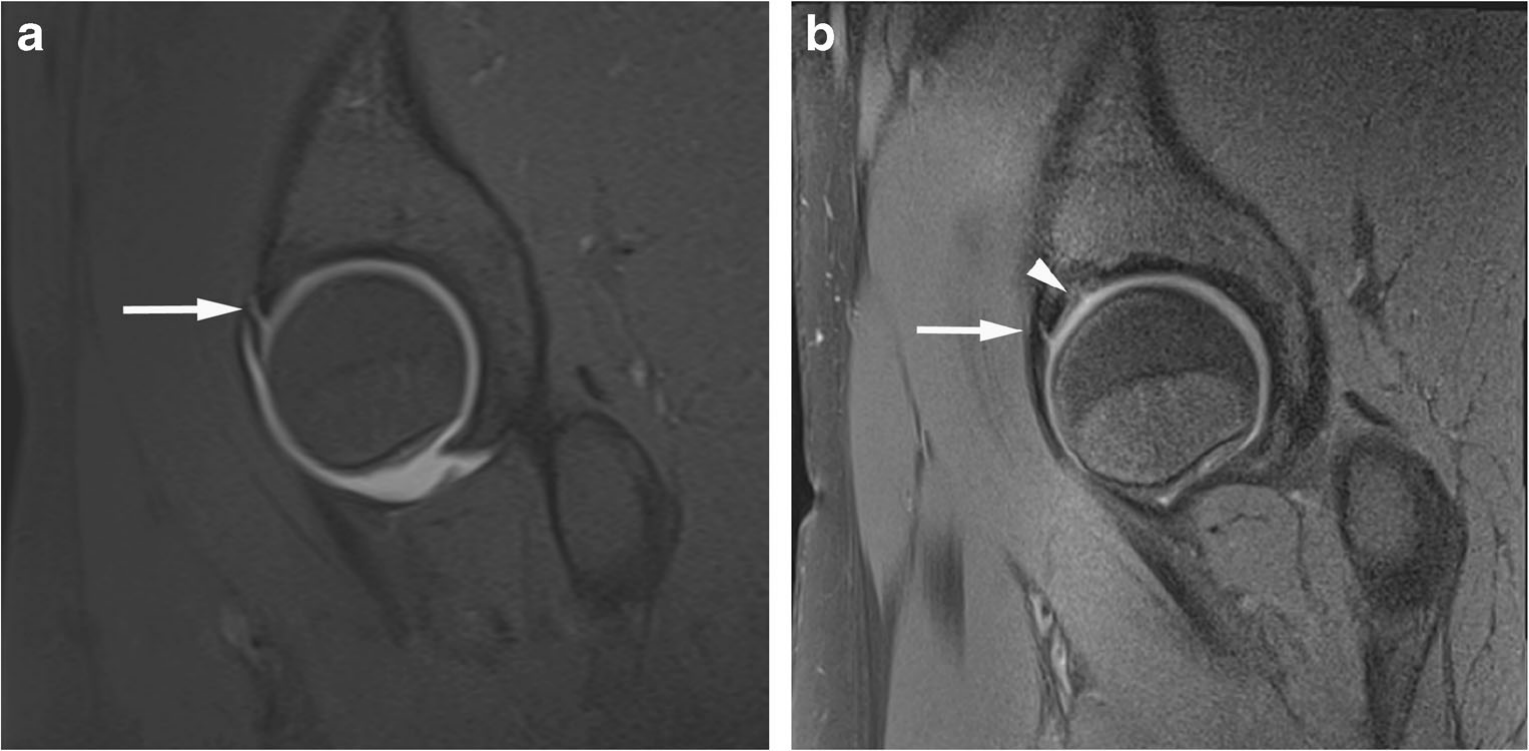
Anterosuperior acetabular labrum complete tear with adjacent full thickness cartilage defect in a 26-year-old confirmed at surgery. Sagittal (**a**) T1 FS 1.5T MRA shows complete basal labral tear (arrow) and normal articular cartilage and (**b**) PD FS 3T MR image shows complete basal labral tear (arrow) and full thickness cartilage defect (arrowhead)

**Fig. 5:**
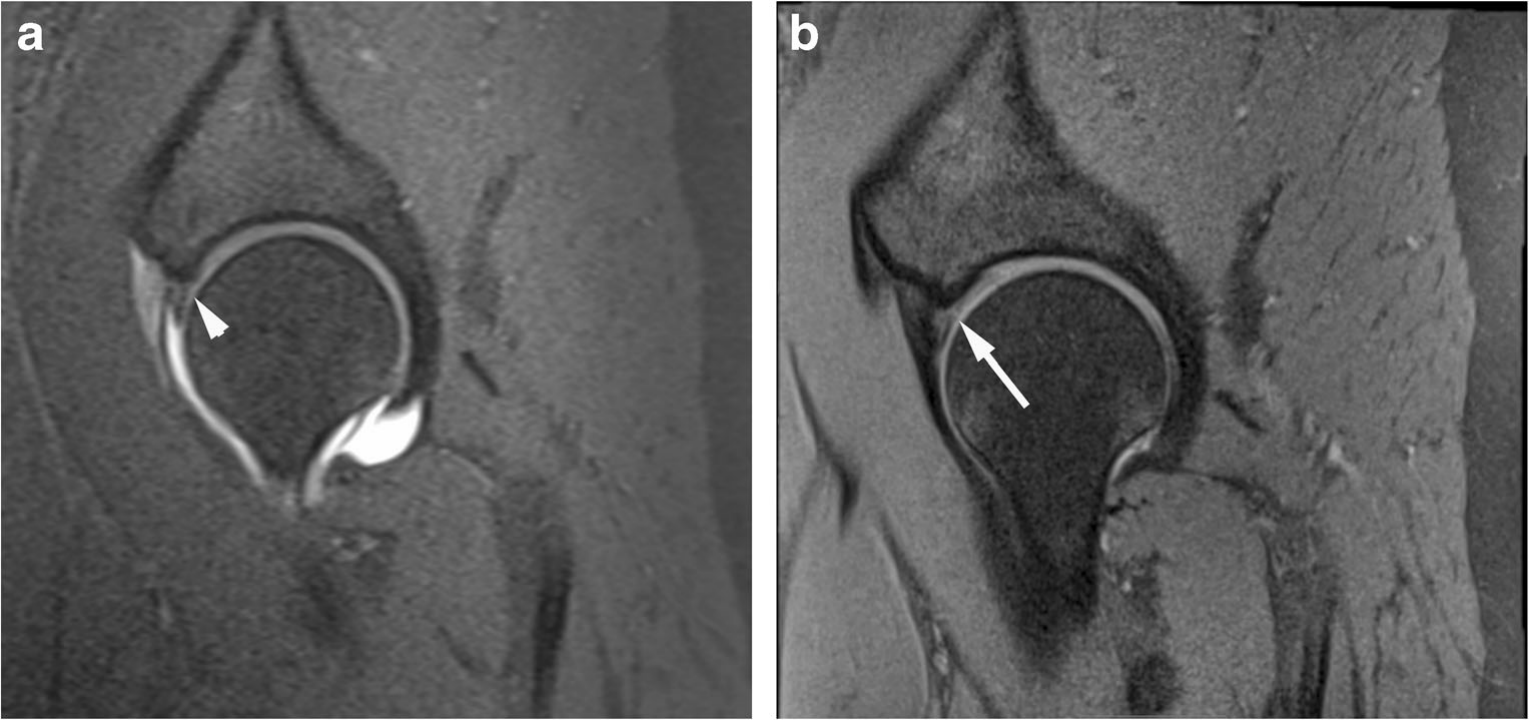
Anterosuperior partial thickness cartilage defect in a 32-year-old confirmed at surgery. Sagittal (**a**) T1 FS 1.5T MRA shows normal articular cartilage at the junction with the labrum (arrowhead) and (**b**) PD FS 3T MR image shows partial thickness cartilage defect (arrow)

**Fig. 6:**
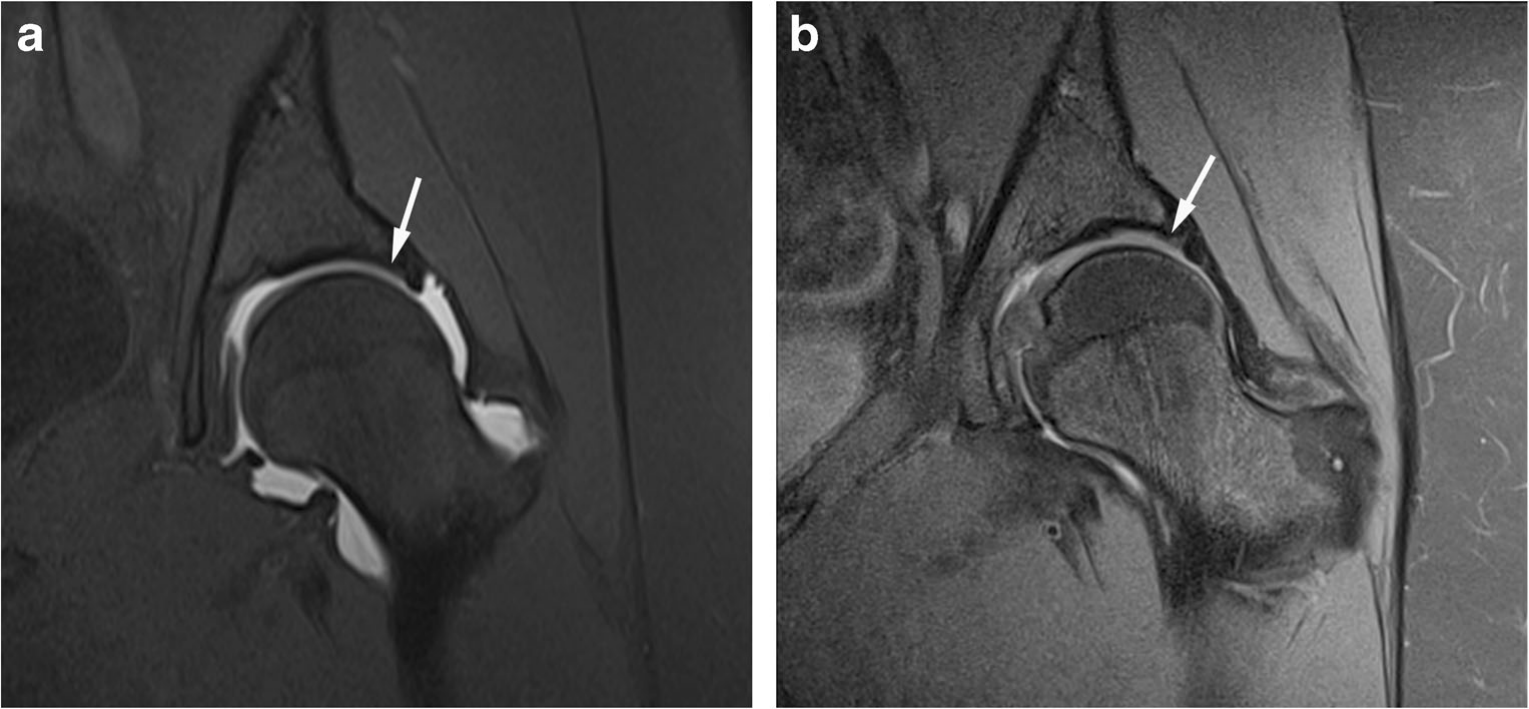
Superior acetabular cartilage delamination in a 31-year-old confirmed at surgery. Coronal (**a**) T1 FS 1.5T MRA image shows intact superior labrum and the adjacent cartilage was scored by both readers as normal. (**b**) PD FS 3T MR image shows intact superior labrum and the adjacent cartilage was scored by both readers as low T1 signal indicating delamination

All anonymised MR images were independently and prospectively analysed by two experienced MSK radiologists (authors 2 and 8, 17 years’ experience each). The radiologists were unaware of whether the patient proceeded to surgery, the surgical findings and the total proportion of patients who underwent surgery. All 1.5T MRA and conventional 3T MRI examinations were evaluated in a random order by each radiologist without reference to the other examination. Each radiologist completed a score sheet evaluating each acetabular quadrant (anterosuperior, posterosuperior, posteroinferior and anteroinferior) for the acetabular labrum (normal, partial tear, full thickness tear) (Figs. 1, 2, 3 and 4), articular cartilage defect (acetabulum and femoral head; normal, partial, full thickness), articular cartilage delamination (present or absent, linear high (fluid) signal intensity on PD or T2 weighted sequences or prominent linear low signal intensity paralleling the subchondral bone plate within/deep to acetabular articular cartilage on T1 or PD weighted sequences) (Figs. 1, 3, 4, 5 and 6) and subchondral oedema (present or absent). Evaluation also included ligamentum teres and transverse ligament (intact or torn), as well as radial femoral bump, femoral pit and acetabular retroversion, recording them as present or absent [15, 16].

### Surgery

Surgeons were blinded to the conventional 3T MRI findings but for ethical reasons were not blinded to 1.5T MRA results. After informed clinical consultation and review, 39/68 study patients subsequently underwent a hip arthroscopy using a standardised technique, performed by an experienced arthroscopist (authors 6 and 7) [17]. At surgery the acetabular labrum was evaluated in each quadrant and scored as normal, partial tear, complete tear and/or degenerate with the position of abnormality recorded. Cartilage was scored as normal, partial thickness defect (< 50 %), full thickness defect or delamination. The position and integrity of the ligamentum teres, transverse ligament and femoral head morphology (including bumps and pits) were recorded.

### Statistical analysis

Statistical analysis was conducted using Stata 13.1 software (StataCorp LP, College Station, TX, USA) and WINPEPI version 11. The overall proportions of exact agreement between the two radiologists were evaluated to determine the exact scoring for 1.5T MRA and conventional 3T MRI separately. Cohen’s kappa and weighted kappa statistics were calculated to evaluate the interobserver agreement using the benchmarks of Landis and Koch: ≤ 0.2 (poor agreement); 0.21–0.40 (fair agreement); 0.41–0.60 (moderate agreement); 0.61–0.8 (substantial agreement); 0.81 (perfect agreement) [18]. Given that kappa may be affected by bias and the imbalance between prevalence of responses, prevalence-adjusted-bias–adjusted-kappa (PABAK) was also calculated and reported [19].

For MRI findings, using arthroscopy as the gold standard the sensitivity and specificity of the two imaging techniques was compared using a McNemar test or exact McNemar test, as appropriate [20]. Given that not all patients underwent arthroscopy, the ordinary estimates of sensitivity and specificity are subject to verification bias. We thus reported Begg and Greenes estimates of sensitivity and specificity, which are corrected for verification bias using a Bayes Theorem approach [21]. To compare diagnostic accuracy between 1.5T MRA and conventional 3T MRI, we applied the methods of Hawass [22], which adjust for the difference in the cells where there was disagreement. Within the same patients, both 1.5T MRA and conventional 3T MRI were compared to a common surgery ‘gold standard’.

## Results

### Patient demographics, questionnaire and surgery findings

A total of 68 participants underwent imaging with both 1.5T MRA and 3T MRI with a median age of 32 years (interquartile range (IQR) 25.5–40.5) and 56 % were female. From this group, 39 participants underwent surgery, with a median age of 34 years (IQR 25–40) and 59 % were female. The results were used to compare the diagnostic accuracy between 1.5T MRA and 3T MRI.

The HOOS questionnaire scores of symptomatic patients proceeding to surgery and those treated non-surgically showed a statistically significant difference, with patients proceeding to surgery having higher HOOS pain, activity, recreation (and sport) and symptom scores (p<0.05). The MMH questionnaires showed no significant difference between the two groups.

At surgery partial labrum tears were observed in nine individuals (23 %) and full thickness tears in 30 (77 %). Twelve (31 %) patients were found to have cartilage delamination and a total of 38 (97 %) patients had acetabular cartilage defect (Table 1). In addition, one ligamentum teres tear and 14 femoral head bump morphologies were recorded. No transverse ligament tears were present. The majority of acetabular pathology scored at surgery involved the anterosuperior quadrant with no abnormality scored in the antero- or posteroinferior quadrants. In all cases where pathology was scored in the posterosuperior quadrant, the same pathology was also scored in the anterosuperior quadrant.

**Table 1:** Summary of surgical findings in test group, n=39

Labrum pathology	All quadrants* N (%)	AS quadrant	PS quadrant
Delamination	12 (31)	12 (31)	2 (5)
Labrum deformation	22 (56)	22 (56)	3 (8)
Labrum ossification	6 (15)	6 (15)	1 (2)
Ligamentum teres (tear)	1 (2)	1 (2)	1 (2)
Acetabular cartilage loss			
Normal	1 (2)	1 (2)	31 (79)
Partial	19 (49)	19 (49)	3 (8)
Full thickness	19 (49)	19 (49)	5 (13)
Femoral cartilage loss			
Normal	32 (82)	32 (82)	39 (97)
Partial	4 (10)	4 (10)	1 (2)
Full thickness	3 (8)	3 (8)	0 (0)
Labrum tear			
Normal	0 (0)	0 (0)	35 (90)
Partial	9 (23)	9 (23)	2 (5)
Full thickness	30 (77)	30 (77)	2 (5)
Femoral bump	13 (33)	13 (33)	1 (2)
Loose bodies	0 (0)	0 (0)	0 (0)

*AS* anterosuperior, *PS* posterosuperior, *AI* anteroinferior, *PI* posteroinferior

*Surgical pathology was only recorded in the AS and PS quadrants and not in the inferior quadrants

### Image analysis

PABAK interobserver agreement for 1.5T MRA and 3T MRI showed substantial to perfect agreement and agreement was similar between the two techniques (Tables 2 and 3). The exception was for cartilage delamination where agreement was moderate using 3T MRI (PABAK = 0.59) compared to substantial agreement at 1.5T MRA (0.79). All other scored parameters (femoral cartilage, ligament teres, transverse ligament, etc) were substantial to perfect (0.81–0.98) except for femoral head morphology, which was moderate (0.55) at 3T and substantial (0.79) at 1.5T MRA.

**Table 2:** Interobserver agreement between radiologists using magnetic resonance arthrography*

	PABAK	PEA % (n/N)	Proportions of category-specific agreement (n/N)		
Dichotomous (present/absent)			Absent	Present	
Rad1 vs. Rad2					
Cyst	0.79	90 (61/68)	94 (106/113)	70 (16/23)	
Delamination	0.79	90 (61/68)	94 (104/111)	72 (18/25)	
lowT1	0.62	81 (55/68)	82 (58/71)	80 (52/65)	
Labrum deformed	0.62	81 (55/68)	48 (34/71)	85 (76/89)	
Labrum ossifcn.	0.85	93 (62/68)	96 (118/123)	55 (6/11)	
Fem. bump	0.79	90 (61/68)	92 (84/91)	84 (38/45)	
Ligamentum teres	0.94	97 (66/68)	98 (124/126)	80 (8/10)	
Pit	0.94	97 (66/68)	99 (128/130)	67 (4/6)	
Ordered categories			Normal	Partial	Full-thickness
Fem. cart loss	0.93	94 (64/68)	97 (122/126)	75 (6/8)	0 (0/2)
Acetabular cart loss	0.82	74 (50/68)	82 (64/78)	53 (18/34)	75 (18/24)
Labrum finding	0.73	68 (46/68)	69 (20/29)	32 (8/25)	78 (64/82)

*PEA* percentage exact agreement

*Quadrant data combined

**Table 3:** Interobserver agreement between radiologists using 3T*

Dichotomous (present/absent)	PABAK	PEA % (n/N)	Proportions of category-specific agreement (n/N)		
			Absent	Present	
Rad1 vs. Rad2					
Cyst	0.68	84 (57/68)	88 (84/95)	73 (30/41)	
Delamination	0.59	79 (54/68)	85 (80/94)	67 (28/42)	
lowT1	0.38	69 (47/68)	43 (16/37)	79 (78/99)	
Labrum deformed	0.59	79 (54/68)	78 (50/64)	81 (58/72)	
Labrum ossifcn.	0.82	91 (62/68)	95 (122/128)	25 (2/8)	
Fem. bump	0.53	77 (52/68)	77 (52/68)	77 (52/68)	
Ligamentum teres	0.79	90 (61/68)	94 (118/125)	36 (4/11)	
Pit	0.97	99 (67/68)	99 (122/123)	92 (12/13)	
Ordered categories			Normal	Partial	Full-thickness
Fem. cart loss	0.90	90 (61/68)	94 (112/119)	62 (8/13)	50 (2/4)
Acetabular cart loss	0.88	75 (51/68)	75 (38/51)	72 (44/61)	83 (20/24)
Labrum finding	0.79	81 (55/68)	36 (4/11)	33 (4/12)	90 (102/113)

*PEA* percentage exact agreement

*Quadrant data combined

Abnormality was only scored by both observers in the anterosuperior and posterosuperior quadrants for MRA and in the anterosuperior, anteroinferior and posterosuperior quadrants at 3T. When analysing agreement separately for each quadrant, there was maintenance of PABAK scores for the anterosuperior and posterosuperior quadrants of substantial to perfect for acetabular and femoral cartilage defect (anterosuperior 0.82–0.93, posterosuperior 0.93–0.99), delamination (anterosuperior 0.62–0.79) and labrum abnormality (anterosuperior 0.71–0.79, posterosuperior 0.87–0.93). Anteroinferior scoring was substantial (0.68) for labrum abnormality.

#### Diagnostic performance of 1.5T MRA versus conventional 3T MRI (Table 4)

For the analysis of labral tears only sensitivity values could be calculated as all participants undergoing surgery had an abnormal labrum. The overall sensitivities for detecting labral tears for both readers were higher with conventional 3T MRI (98 %) compared to 1.5T MRA (79–82 %), but these differences were not statistically significant (p=0.07). Both readers had identical detection rates for acetabular cartilage defect with higher overall sensitivities using conventional 3T MRI (84 %) versus 1.5T MRA (61 %), and these differences were statistically significant (p=0.02). The results for cartilage delamination show that Reader 1 had a slightly higher sensitivity and specificity using 1.5T MRA whereas Reader 2 had better sensitivity with conventional 3T MRI but a lower specificity. However, these results were not shown to be statistically significant (p=0.66). Other parameters (femoral cartilage, ligament teres and transverse ligament also showed variable sensitivities (9–100 %) and high specificities (> 89–100 %), but in areas with a very low incidence of abnormality, and figures were not statistically significant.

**Table 4:** Comparison of diagnostic accuracy between magnetic resonance arthrography (MRA) and 3T^1^

	Labral tear(n)	Sensitivity (%)	Specificity (%)	Acetabular cartilage defect (n)	Sensitivity (%)	Specificity (%)	Cartilage delamination (n)	Sensitivity (%)	Specificity (%)
Reader 1 MRA									
AS quadrant	32	82	n/a	21	55*	100	5	42	82
PS quadrant	1	25	97	1	14	100	0	0	97
Reader 2 MRA									
AS quadrant	31	79	n/a	17	45*	100	3	25	89
PS quadrant	1	25	94	1	20	97	0	0	97
Reader 1 3T									
AS quadrant	36	92	n/a	30	79*	100	4	33	78
PS quadrant	3	75	89	2	29	78	0	0	97
AI quadrant	n/a	n/a	97	0	0	n/a	n/a	n/a	100
Reader 2 3T									
AS quadrant	35	90	n/a	27	71*	100	4	33	74
PS quadrant	1	25	89	3	43	81	0	0	97
AI quadrant	n/a	n/a	90	0	0	n/a	n/a	n/a	100

*AS* anterosuperior, *PS* posterosuperior, *AI* anteroinferior, *PI* posteroinferior

*Statistically significant difference between MRA and 3T (p=0.02)

^1^No MRI pathology was recorded in the AI and PI quadrants at MRA. No MRI pathology was recorded in the PI quadrant at 3T

Abnormality was only scored in the anterosuperior and posterosuperior quadrants with only relatively small numbers in the posterosuperior quadrant, so statistical significance could not be accurately evaluated in this quadrant. However, the statistically significant difference for cartilage defect evaluation present in the combined analysis was still evident in the data for the anterosuperior quadrant alone.

## Discussion

Currently the standard approach for imaging FAI is with MR arthrography as it is the modality of choice for evaluating the acetabular labrum and chondral defects [2, 23, 24]. There have been three recently published studies that, like this current study, have directly compared MRA and MRI findings with surgical findings in patients suspected of FAI [6–8]. Although our results showed a diagnostic advantage for detecting labral tears using conventional 3T MRI over 1.5T MRA, these differences were not statistically significant, but suggest at least equivalence between the two techniques. Sutter et al. prospectively reviewed 28 patients who underwent 1.5T MRA and 1.5T MRI and also found no statistically significant difference between the two techniques. Their sensitivities for labral tear detection with conventional 1.5T MRI (77 % and 89 %) were lower than ours at 3T MRI (90–92 %), a difference that may be accounted for by the difference in field strength utilised in the two studies. Tian et al. retrospectively compared conventional 3T MRI with 3T MRA in 21 patients with surgically proven labral tears and found statistically significant differences in favour of 3T MRA with sensitivities of 95 % for 3T MRA versus 66 % for conventional 3T MRI [8]. However, another more recent retrospective study of 43 patients by Magee, showed equivalent accuracy for the detection of labral tears between 3T MRA and conventional 3T MRI for both readers [6].

The detection of acetabular cartilage defects poses a specific diagnostic challenge, where MR arthrography is believed to have a potential advantage [16]. We report a statistically significant difference for conventional 3T MRI and1.5T MRA cartilage defect detection in the anterosuperior quadrant; however, this apparent superiority should be treated cautiously as for other pathologies both techniques predominantly showed equivalence. Magee’s study (3T MRI vs. 3T MRA) showed superior cartilage defect detection for 3T MRA that was not statistically significant. While our study would suggest that there is an advantage in using 3T MRI over 1.5T MRA, Magee’s study indicates the addition of intra-articular contrast at 3T may offer a further advantage, although more studies would be required to determine whether this reaches statistical significance. Sutter et al. found an increased accuracy for detecting acetabular chondral defects with 1.5T MRA compared to conventional 1.5T MRI for both readers [7], while Tian et al. did not evaluate the results for chondral defects [8].

Recognising acetabular cartilage delamination is important as joint-preserving surgery can be attempted leading to symptomatic relief and improved prognosis [25]. Detection often relies on the presence of a fluid cleft between the cartilage and subchondral plate. This can be challenging due to the closely opposed articular surfaces which can effectively ‘close off’ the cleft, and it is thought arthrographic contrast should improve the detection by contrast flowing into this deep layer. Pfirrmann et al. retrospectively evaluated 1.5T MRA for cartilage delamination [16] and found that a fluid cleft was specific but insensitive for delamination, but hypointensity of articular cartilage on intermediate weighted fat-saturated and T1 sequences could be a helpful indicator of delamination (sensitivity 74 % and specificity 90 % for their most experienced reader). Conversely, Linda et al. found discordance between conventional 3T MRI and surgical findings for the assessment of cartilage delamination to be more marked than for other features of chondral damage [5]. We also found a low overall detection rate for both readers, evaluating for fluid and/or chondral hypointensity with low sensitivities using 1.5T MRA and conventional 3T MRI with better interobserver agreement for 1.5T MRA, but no statistically significant difference between the two (Table 4, Figs. 3 and 6).

Linda et al. demonstrated better sensitivity and specificity than we achieved at 3T MRI for labral and chondral pathology [5], although this was a retrospective study and readings were made by consensus. Compared to the current study, there were also significant differences in the 3T MRI protocol used, most notably the use of radial imaging sequences. Radial sequences have not been formally assessed to determine the additional benefit they may offer, but we recognise that there may be improvements that could be made to our protocol, including the addition of radial sequences, which may further improve the accuracy of this technique. It would also be possible to achieve improvements in signal-to-noise ratio and resolution, although a time penalty would be incurred. A similar argument could apply to refining the MRA examination. In this study we aimed to utilise protocols that were already in clinical use, using manufacturers’ sequences that would achieve imaging times practical for clinical use. It would have been easy to improve one protocol at the expense of the other by making it longer. As a result, we were particularly concerned to ensure that the two protocols used were of similar duration, in this case both under 30 min.

Another recent study utilising 1.5T MRA has suggested that the diagnosis of chondral and labral pathology in the hip might be improved by the use of traction on the leg. This is not an area we have explored, but it is interesting to note that the sensitivity to labral tears reported in the study using traction is similar to the sensitivities we report for 3T MRI [26].

As with all the other studies discussed, the current study was limited by the relatively small study size and the assumption that the surgical findings at arthroscopy were the gold standard. The two radiologists in our study were completely blinded to the results of the arthroscopy and the proportion proceeding to surgery, and the images were prospectively interpreted in a random order. However, there is a risk of inevitable detection bias towards a largely symptomatic patient population. Future developments are possible, including the evaluation of 3T MRA, leg traction and new orientation sequences, which may or may not add diagnostic benefit. These were not specifically evaluated in this study, in an effort to keep imaging times practical and similar between the two protocols.

In conclusion, the results of this study show that 3T MRI is at least equivalent to 1.5T MRA for detecting acetabular labral tears and cartilage abnormality in patients with suspected femoroacetabular impingement.

## Abbreviations

MRA: Magnetic resonance arthrography
MRI: Magnetic resonance imaging
1.5T and 3T: 1.5 Tesla and 3 Tesla

## References

[1] Wagner S, Hofstetter W, Chiquet M (2003). Early osteoarthritic changes of human femoral head cartilage subsequent to femoro-acetabular impingement. Osteoarthr Cartil.

[2] Smith TO, Hilton G, Toms AP, Donell ST, Hing CB (2011). The diagnostic accuracy of acetabular labral tears using magnetic resonance imaging and magnetic resonance arthrography: a meta-analysis. Eur Radiol.

[3] James SL, Ali K, Malara F, Young D, O'Donnell J, Connell DA (2006). MRI findings of femoroacetabular impingement. Am J Roentgenol.

[4] Mintz DN, Hooper T, Connell D, Buly R, Padgett DE, Potter HG (2005). Magnetic resonance imaging of the hip: detection of labral and chondral abnormalities using noncontrast imaging. Arthroscopy.

[5] Linda DD, Naraghi A, Murnaghan L, Whelan D, White LM (2016) Accuracy of non-arthrographic 3T MR imaging in evaluation of intra-articular pathology of the hip in femoroacetabular impingement. Skelet Radiol:1-10 10.1007/s00256-016-2551-z 27975135

[6] Magee T (2015). Comparison of 3.0-T MR vs 3.0-T MR arthrography of the hip for detection of acetabular labral tears and chondral defects in the same patient population. Br J Radiol.

[7] Sutter R, Zubler V, Hoffmann A (2014). Hip MRI: how useful is intraarticular contrast material for evaluating surgically proven lesions of the labrum and articular cartilage?. AJR Am J Roentgenol.

[8] Tian CY, Wang JQ, Zheng ZZ, Ren AH (2014). 3.0 T conventional hip MR and hip MR arthrography for the acetabular labral tears confirmed by arthroscopy. Eur J Radiol.

[9] Lee AJ, Armour P, Thind D, Coates MH, Kang AC (2015). The prevalence of acetabular labral tears and associated pathology in a young asymptomatic population. Bone Joint J.

[10] Harris WH (1969). Traumatic arthritis of the hip after dislocation and acetabular fractures: treatment by mold arthroplasty. An end-result study using a new method of result evaluation. J Bone Joint Surg Am.

[11] Klassbo M, Larsson E, Mannevik E (2003). Hip disability and osteoarthritis outcome score. An extension of the Western Ontario and McMaster Universities Osteoarthritis Index. Scand J Rheumatol.

[12] Nilsdotter AK, Lohmander LS, Klassbo M, Roos EM (2003). Hip disability and osteoarthritis outcome score (HOOS)--validity and responsiveness in total hip replacement. BMC Musculoskelet Disord.

[13] Zahiri CA, Schmalzried TP, Szuszczewicz ES, Amstutz HC (1998). Assessing activity in joint replacement patients. J Arthroplast.

[14] Llopis E, Fernandez E, Cerezal L (2012). MR and CT arthrography of the hip. Semin Musculoskelet Radiol.

[15] Schmid MR, Notzli HP, Zanetti M, Wyss TF, Hodler J (2003). Cartilage lesions in the hip: diagnostic effectiveness of MR arthrography. Radiology.

[16] Pfirrmann CW, Duc SR, Zanetti M, Dora C, Hodler J (2008). MR arthrography of acetabular cartilage delamination in femoroacetabular cam impingement. Radiology.

[17] Espinosa N, Beck M, Rothenfluh DA, Ganz R, Leunig M (2007). Treatment of femoro-acetabular impingement: preliminary results of labral refixation. Surgical technique. J Bone Joint Surg Am.

[18] Landis JR, Koch GG (1977). The measurement of observer agreement for categorical data. Biometrics.

[19] Byrt T, Bishop J, Carlin JB (1993). Bias, prevalence and kappa. J Clin Epidemiol.

[20] Lachenbruch PA, Lynch CJ (1998). Assessing screening tests: extensions of McNemar's test. Stat Med.

[21] Begg CB, Greenes RA (1983). Assessment of diagnostic tests when disease verification is subject to selection bias. Biometrics.

[22] Hawass NE (1997). Comparing the sensitivities and specificities of two diagnostic procedures performed on the same group of patients. Br J Radiol.

[23] Kassarjian A (2006). Hip MR arthrography and femoroacetabular impingement. Semin Musculoskelet Radiol.

[24] Kassarjian A, Yoon LS, Belzile E, Connolly SA, Millis MB, Palmer WE (2005). Triad of MR arthrographic findings in patients with cam-type femoroacetabular impingement. Radiology.

[25] Clohisy JC, McClure JT (2005). Treatment of anterior femoroacetabular impingement with combined hip arthroscopy and limited anterior decompression. Iowa Orthop J.

[26] Schmaranzer F, Klauser A, Kogler M (2015). Diagnostic performance of direct traction MR arthrography of the hip: detection of chondral and labral lesions with arthroscopic comparison. Eur Radiol.

